# A switchable [2]rotaxane with two active alkenyl groups

**DOI:** 10.3762/bjoc.14.181

**Published:** 2018-08-08

**Authors:** Xiu-Li Zheng, Rong-Rong Tao, Rui-Rui Gu, Wen-Zhi Wang, Da-Hui Qu

**Affiliations:** 1 Key Laboratory for Advanced Materials and Institute of Fine Chemicals, School of Chemistry and Molecular Engineering, East China University of Science and Technology, 130 Meilong Road, Shanghai, 200237, China

**Keywords:** alkenyl bond, functional crown ether, stimuli-responsiveness, switchable rotaxane

## Abstract

A novel functional [2]rotaxane containing two alkenyl bonds was designed, synthesized and characterized by ^1^H, ^13^C NMR spectroscopy and HRESI mass spectrometry. The introduction of alkenyl bonds endowed the [2]rotaxane a fascinating ability to react with versatile functional groups such as alkenyl and thiol functional groups. The reversible shuttling movement of the macrocycle between two different recognition sites on the molecular thread can be driven by external acid and base. This kind of rotaxane bearing functional groups provides a powerful platform for preparing stimuli-responsive polymers.

## Introduction

Along with the development of supramolecular chemistry, much attention has been paid to the design and synthesis of novel and complicated mechanical interlocked molecules (MIMs) [1–7]. During the past years, a large number of different MIMs has been constructed, such as handcuff catenane [8], molecular elevators [9–10], molecular muscles [11–12], trefoil necklace [13–15], molecular walkers [16–18] and molecular pumps [19–20]. Combining the stimuli-responsive microscopic units with traditional materials to achieve morphological changes or other novel properties in smart materials is continuously receiving wide attention. Hence, in recent years, scientists have put more interests on the macroscopic changes caused by the stimuli-responsiveness of constituent units, which act as important precursors for constructing stimuli-responsive supramolecular materials [21–22].

Rotaxanes [23–31], as one of the most important MIMs, have been deeply investigated because of their excellent properties and convenient synthesis. By introducing various functional groups, such kind of MIMs has been used to construct stimuli-responsive materials, which diversified and improved the functions of traditional polymers. Up to now, the repertoire of available functional rotaxanes as building blocks for the fabrication of stimuli-responsive polymers remains limited because of the fact that the induction of active and functional groups make the preparation of rotaxanes more difficult and complicated. Therefore, it is urgent to enrich both the family of stimuli-responsive units and the methods for constructing this kind of smart materials.

In this paper, we report the design, synthesis, characterization and shuttling motion of a [2]rotaxane **R1**, which is modified with two naked alkenyl bonds. In this [2]rotaxane, a functional dibenzo-24-crown-8 (DB24C8) macrocycle is interlocked with an flexible chain and could perform reversible shuttling between two different recognition sites under the stimuli of external acid and base. The alkenyl bonds were chosen as the functional group due to their considerable stability during the synthesis and remarkable reaction activity. The latter indicates the ability of reacting with different functional groups such as alkenyl bonds in the presence of Grubbs’ catalyst and thiol groups irradiated by UV light. Besides, the shuttling movement of the DB24C8 macrocyclic ring was confirmed by ^1^H NMR spectroscopy.

## Results and Discussion

**Scheme 1:**
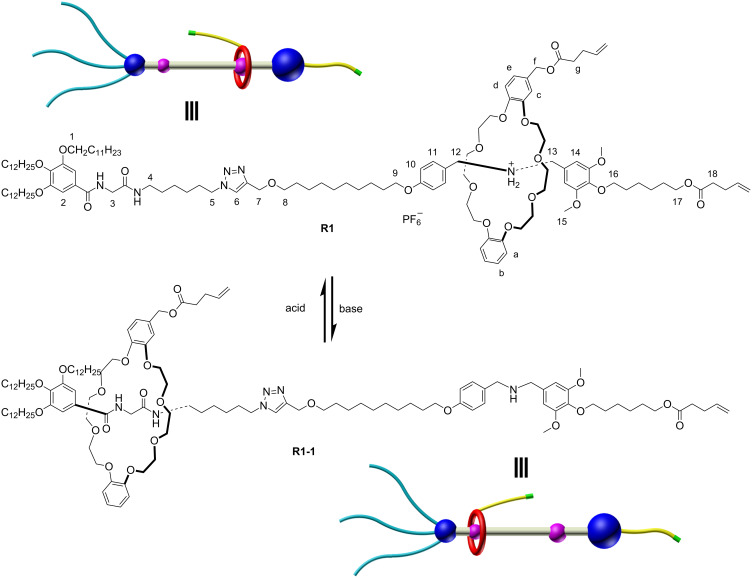
Chemical structures and acid-base stimuli responsiveness of target [2]rotaxane **R1** and deprotonated [2]rotaxane **R1-1**.

The structures of the two states of [2]rotaxane **R1** are shown in Scheme 1. In the target structure [2]rotaxane **R1**, the DB24C8 macrocycle is functionalized with an alkenyl unit on one of the benzene groups. Two distinguishable recognition sites, a dibenzylammonium (DBA) and an amide binding site, are introduced to the axle and linked with a long alkyl chain. The amide moiety could act as another combining station when the DBA site is deprotonated by external base. Besides, in the structure, one side of the chain is terminated by a bulky stopper bearing three long alkyl chains to prevent the macrocyclic moiety from slipping out the thread. Meanwhile, the long alkyl chains make it easy to form gels when the alkenyl units are reacted to generate polymers. On the other side, a naked alkenyl bond is introduced in the *para* position of the aromatic stopper. In the presence of external acid–base stimuli, the macrocyclic ring could be driven back and forth along the linear thread. Deprotonation of the DBA site by base makes the DB24C8 moiety slide to the amide station and the macrocycle could move back to the DBA recognition site when acid was added (Scheme 1).

**Scheme 2:**
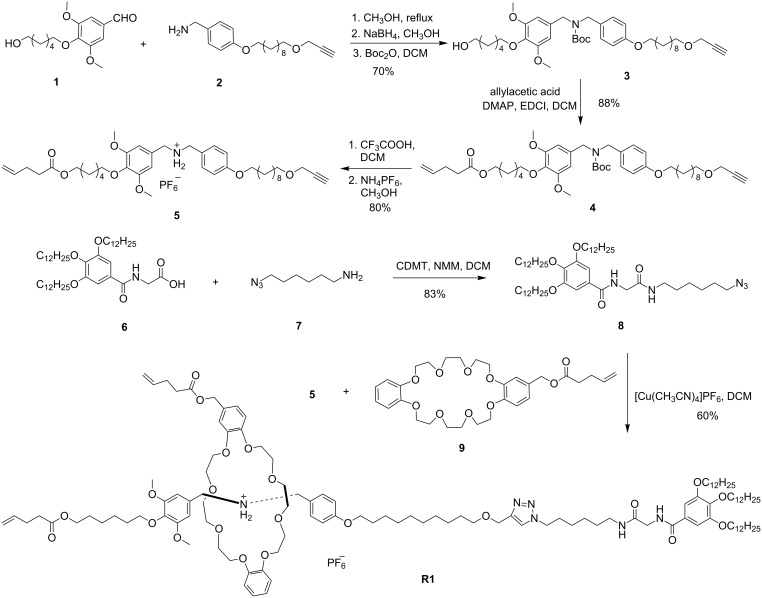
Syntheses of key intermediates **5**, **8** and target [2]rotaxane **R1**.

The synthesis routes of the target compound [2]rotaxane **R1** are shown in Scheme 2. Compounds **1** [32], **2** [33], **6** [34], **7** [35] and functional crown ether **9** [36] were synthesized according to the previous literature. The key intermediate compound **5**, containing a prior DBA recognition station and possessing alkynyl and ethylenic groups at two terminals respectively, was prepared from aldehyde **1** and amine **2**. A “Schiff base” reaction was introduced at first and treated with NaBH_4_, protected by di-*tert*-butyl pyrocarbonate to get compound **3**. The esterification reaction was carried out subsequently between hydroxy compound **3** and allylacetic acid to afford compound **4**, which was further reacted with CF_3_COOH and followed by the anion exchange in the methyl alcohol with saturated NH_4_PF_6_ solution to obtain compound **5**. For another key intermediate **8**, containing an amide site for stabilizing the DB24C8 macrocycle, a 1,2,3-tris(dodecyloxy) benzene group as a stopper and an azide group which is used for the reaction with other moieties, was prepared through the amide reaction between compound **6** and 6-azidohexan-1-amine in the presence of 2-chloro-4,6-dimethoxy-1,3,5-triazine and *N*-methylmorpholine. Finally, the classical and effective “Click” reaction was used to produce the target compound [2]rotaxane **R1**. The crown ether **9** and dibenzylammonium ion (R_2_NH_2_^+^) containing intermediate compound **5** were assembled in dichloromethane through host–guest interaction and capped with compound **8** under Cu(I)-catalyzed azide–alkyne cycloaddition to get the final [2]rotaxane with two distinguishable recognition sites.

**Figure 1:**
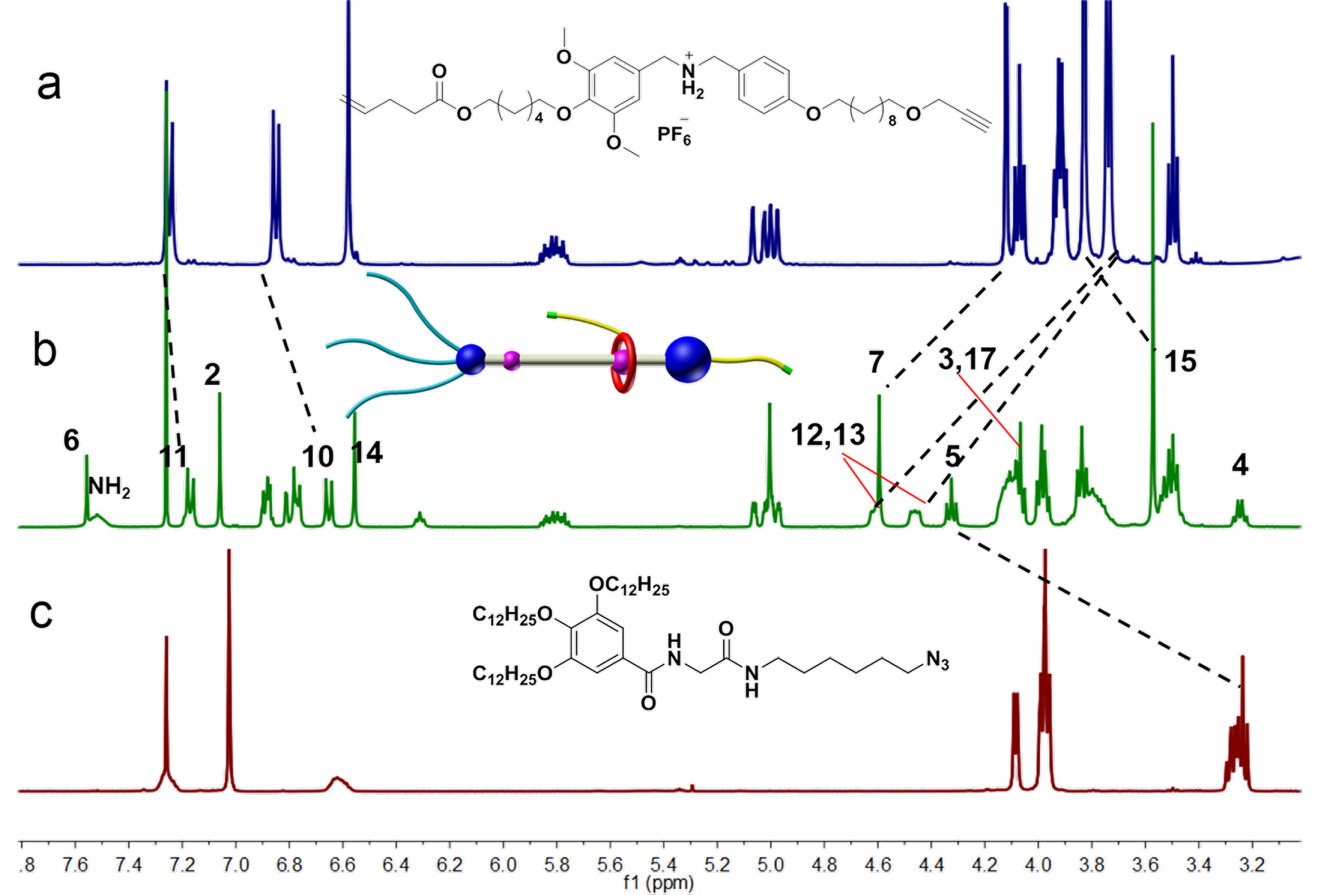
Partial ^1^H NMR spectra (400 MHz, CDCl_3_, 298 K). (a) Compound **5**, (b) target [2]rotaxane **R1**, (c) azide compound **8**.

The target [2]rotaxane **R1** was then characterized by ^1^H NMR spectroscopy and high-resolution electrospray ionization (HRESI) mass spectrometry. The HRESI mass spectra of [2]rotaxane **R1** showed a major peak at *m*/*z* 2083.3862, corresponding to the species of **R1** losing one PF_6_^−^ anion, i.e., [M − PF_6_^−^]^+^. The ^1^H NMR spectrum also fitted the **R1** structure well and reveals that the DB24C8 macrocycle **9** prefers encompassing the DBA recognition site. Comparing the ^1^H NMR spectrum of the rotaxane product **R1** with the reactants **8** and **5** (Figure 1), the resonances of protons H5 and H7 were both shifted downfield (Δδ_H5_ = 1.09 ppm and Δδ_H7_ = 0.51 ppm) while a new peak appeared at 7.56 ppm. This new peak corresponds to H6 coming from the alkynyl–azide cycloaddition. Meanwhile, the signal of H12 and H13 split into two signals and underwent downfield shifts (Δδ_H12_ = 0.73 ppm and Δδ_H13_ = 0.87 ppm) while those of H11 and H10 moved upfield (Δδ_H11_ = −0.08 ppm and Δδ_H10_ = −0.20 ppm). Nevertheless, there were no obvious changes in protons of H3, H4 and H2, indicating that the macrocycle stayed at the DBA recognition site. These results were consistent with the previous literature report by our group [37]. All the evidences discussed above demonstrate that the axle compound successfully threaded into the macrocycle and consequently formed the target [2]rotaxane **R1**.

**Figure 2:**
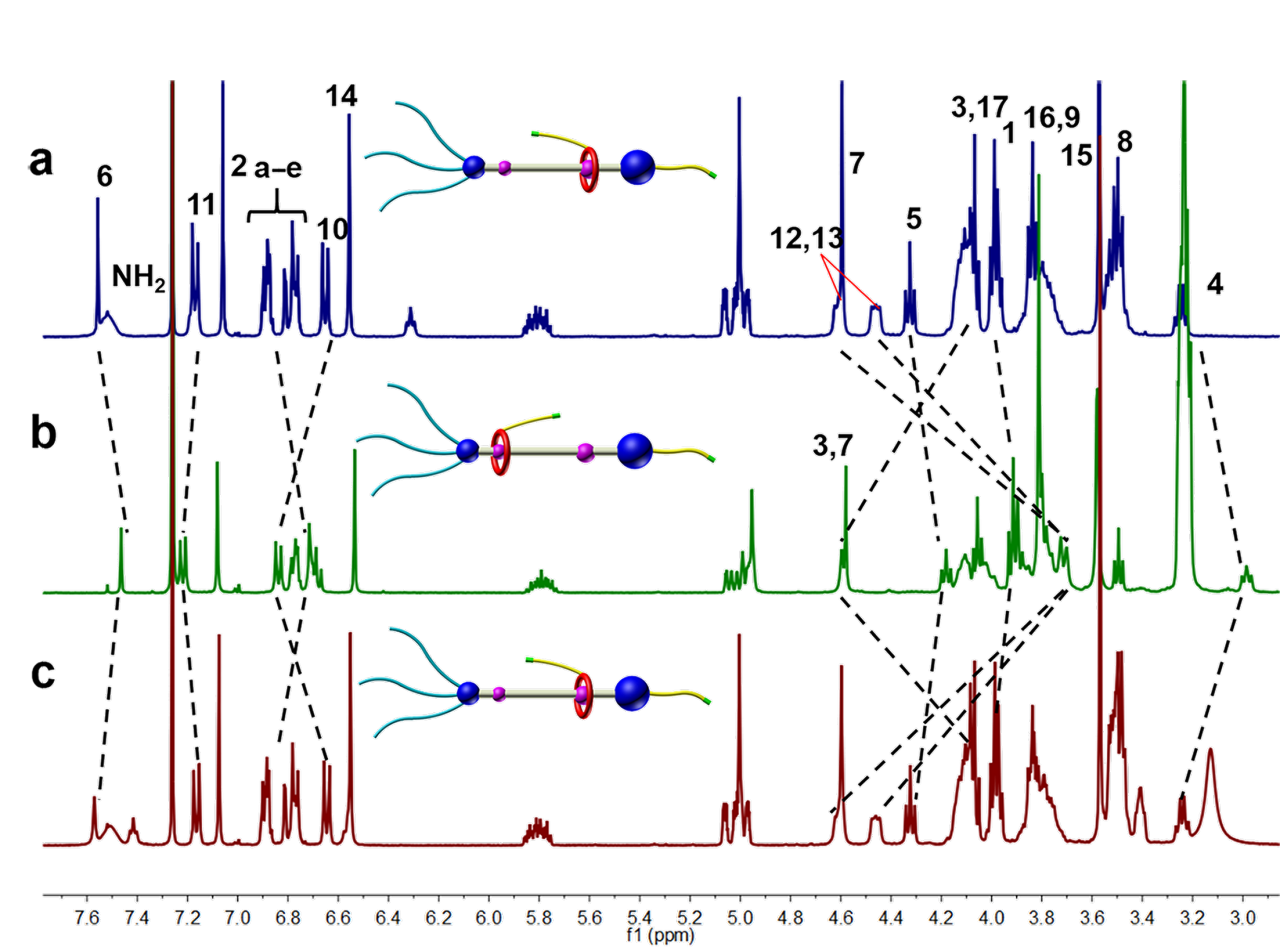
Partial ^1^H NMR spectra (400 MHz, CDCl_3_, 298 K). (a) [2]Rotaxane **R1**, (b) deprotonation by the addition of 2.0 equiv of DBU to (a), (c) reprotonation with addition of 3.0 equiv of TFA to (b).

The reversible shuttling motion of the functional DB24C8 macrocycle between the two different recognition sites on the axle was also confirmed by the ^1^H NMR spectroscopy. After addition of 2 equiv1,8-diazabicyclo[5.4.0]undec-7-ene (DBU) to [2]rotaxane **R1** in CDCl_3_ to deprotonate the ammonium moiety, the DB24C8 macrocycle moved to the amide station. As shown in Figure 2, the protons H12, H13 shifted upfield (Δδ_H12_ = −0.99 ppm and Δδ_H13_ = −0.75 ppm) and incorporated into one signal peak from two while the H11 and H10 shifted slightly downfield (Δδ_H11_ = 0.05 ppm and Δδ_10_ = 0.19 ppm) due to the deprotonation of the R_2_NH_2_^+^ and the migration of macrocycle. At the same time, the peaks for the protons around the amide site changed, for H4, H5, H6 with Δδ_H4_ = −0.26 ppm, Δδ_H5_ = −0.14 ppm and Δδ_H6_ = −0.09 ppm, respectively and H_3_ with a Δδ_H3_ = 0.51 ppm due to the association with the DB24C8 macrocycle through hydrogen bonding. All the evidences reveal that the functionalized macrocycle migrated from the DBA recognition site to the amide station when an external base was added to the solution of rotaxane **R1**. Then, 3 equiv trifluoroacetic acid were added to reprotonate the -NH- moiety, the ^1^H NMR spectrum restored to the initial state, showing that the macrocyclic compound **9** moved back to the DBA recognition site. Therefore, the reversible shuttling movement of DB24C8 moiety along the thread between two recognition sites driven by acid-base stimuli has been confirmed.

**Figure 3:**
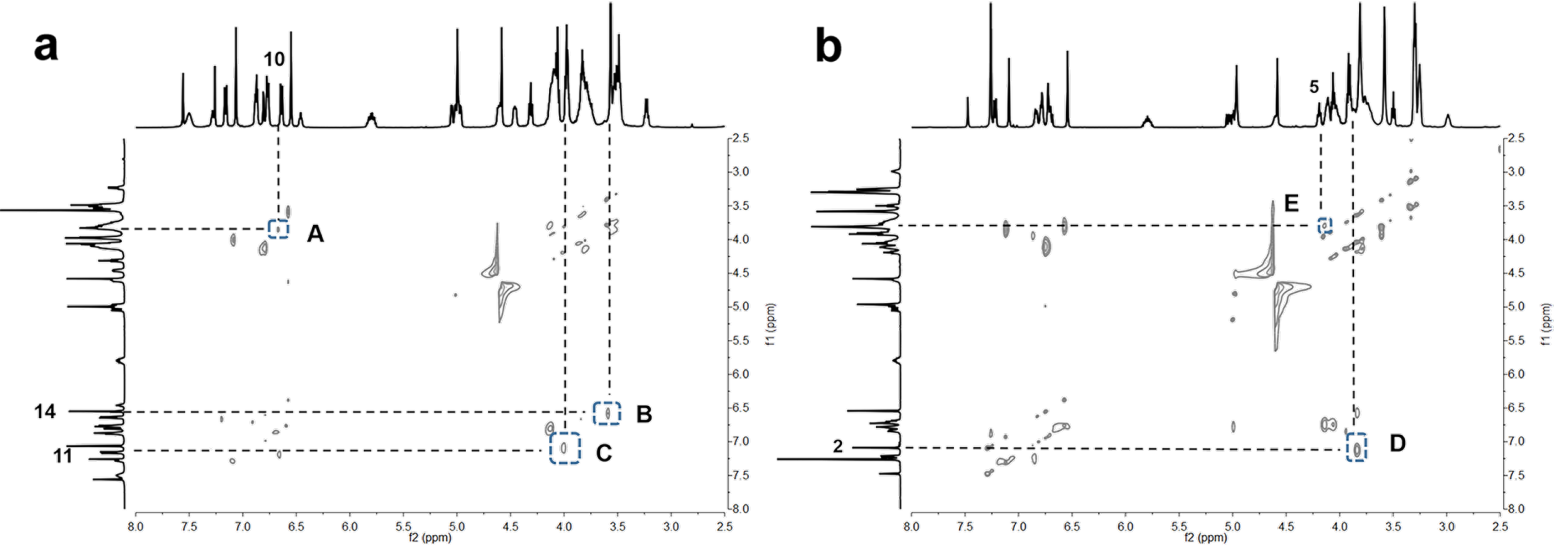
Partial 2D ROESY NMR spectra (500 MHz, CDCl_3_, 298 K). (a) [2]Rotaxane **R1**, (b) deprotonation with addition of 2.0 equiv of DBU to (a).

To further demonstrate the binding mode of the functional macrocycle with the axle, 2D ROESY NMR spectra of rotaxane **R1** before and after the deprotonation in CDCl_3_ were obtained. As shown in Figure 3a, the cross-peaks of phenyl protons H10 (peak A), H14 (peak B), H11 (peak C) (around the DBA recognition site) and methylene protons on the functional crown ether indicate that the DB24C8 macrocycle was located on the DBA site, corresponding to the structure of [2]rotaxane **R1**. After addition of 2 equiv DBU to the solution of **R1**, the NOE correlations between the phenyl proton H2 (peak D) near the amide station, H5 (peak E) on the thread and methylene proton on DB24C8 could be observed (see Figure 3b), illustrating the position of the DB24C8 ring on the amide recognition site.

## Conclusion

In summary, a novel functional [2]rotaxane **R1** with two alkenyl bonds on the DB24C8 macrocycle and axle, respectively, was prepared and well characterized. The shuttling movement of the functionalized DB24C8 macrocycle between the DBA recognition site and amide moiety could be realized by stimuli of external acid-base and was investigated by ^1^H NMR and 2D ROESY NMR spectroscopy. The naked alkenyl groups could react with other functional groups such as alkenyl and thiol groups to prepare stimuli-responsive polymers. Besides, the introduction of long alkyl chains makes the polymers easier to form gels. This kind of functional rotaxane enriches the species of building blocks to construct stimuli-responsive polymers and smart materials.

## Experimental

### General and materials

^1^H NMR and ^13^C NMR spectra were measured on a Bruker AV-400 spectrometer. The electronic spray ionization (ESI) mass spectra were tested on a LCT Premier XE mass spectrometer.

Chemicals were used as received from Acros, Aldrich, Fluka, or Merck. All solvents were reagent grade, which were dried and distilled prior to use according to standard procedures. The molecular structures were confirmed via ^1^H NMR, ^13^C NMR and high-resolution ESI mass spectroscopy. The synthesis of compound **1**, **2**, **6**, **7**, **9** have already been reported.

### Synthesis

**Compound 8:** The mixture of compound **6** (5.0 g, 6.83 mmol), compound **7** (2.9 g, 20.49 mmol) and 2-chloro-4,6-dimethoxy-1,3,5-triazine (CDMT, 3.6 g, 20.49 mmol) was placed in a 250 mL round-bottom flask and dissolved by 100 mL methylene chloride. Then, *N*-methylmorpholine (NMM, 3.5 g, 34.15 mmol) was added into the solution slowly under ice bath cooling. Afterwards, the mixture was stirred at 40 °C for 12 h under an argon atmosphere. The reaction mixture was cooled to room temperature, the solution was evaporated under reduced pressure and the residue was purified via column chromatography (SiO_2_, PE/EA = 3:1) to give compound **8** (4.9 g, 83%) as a white solid. ^1^H NMR (CDCl_3_, 400 MHz, 298 K) δ 7.30–7.23 (m, 1H), 7.02 (s, 2H), 6.67–6.65 (m, 1H), 4.28 (d, *J* = 5.2 Hz, 2H), 4.02–3.93 (m, 6H), 3.31–3.19 (m, 4H), 1.80–1.72 (m, 6H), 1.61–1.49 (m, 4H), 1.48–1.40 (m, 6H), 1.36–1.23 (m, 52H), 0.87 (t, *J* = 6.8 Hz, 9H); ^13^C NMR (CDCl_3_, 100 MHz, 298 K) δ 169.2, 167.8, 153.1, 141.3, 128.1, 105.6, 73.5, 69.2, 51.2, 44.1, 39.5, 31.9, 30.2, 29.70, 29.68, 29.66, 29.62, 29.56, 29.39, 29.36, 29.34, 29.31, 28.7, 26.4, 26.3, 26.07, 26.05, 22.7, 14.1; HRMS–ESI–TOF (*m*/*z*): [M + K]^+^ calcd for C_51_H_93_N_5_O_5_K^+^, 894.6808; found, 894.6813.

**Compound 3:** A mixture of **1** (3.0 g, 10.63 mmol) and **2** (4.0 g, 12.75 mmol) in methyl alcohol (100 mL) was refluxed overnight under a nitrogen atmosphere. After the reaction mixture was cooled to room temperature, NaBH_4_ (1.6 g, 30.5 mmol) was then added to the solution in portions while colling in an ice bath. After the mixture was stirred overnight, the solvent was removed under vacuum, and the residue was extracted by dichloromethane. The organic layer was washed by brine, dried with Na_2_SO_4_, and then concentrated to give the free amine compound. To the solution of the amine in dry DCM (50 mL) was added di-*tert*-butyldicarbonate (3.5 g, 15.95 mmol) and the mixture was stirred for 5 h at room temperature. Then, the reaction mixture was washed with water, dried over Na_2_SO_4_ and evaporated in vacuo to give a crude product, which was purified by column chromatography (SiO_2_, PE/EA = 2:1) to afford product **3** (4.4 g, 60%) as a yellow oil. ^1^H NMR (CDCl_3_, 400 MHz, 298 K) δ 7.20–7.01 (m, 2H), 6.83 (d, *J* = 8.4 Hz, 2H), 6.37 (d, *J* = 26.0 Hz, 2H), 4.29 (dd, *J* = 32.8 Hz, *J* = 18.4 Hz, 4H), 4.12 (s, 2H), 3.97–3.87 (m, 4H), 3.78 (s, 6H), 3.62 (t, *J* = 6.4 Hz, 2H), 3.49 (t, *J* = 6.4 Hz, 2H), 2.41 (s, 1H), 1.79–1.70 (m, 4H), 1.61–1.54 (m, 4H), 1.52–1.35 (m, 15H), 1.37–1.27 (m, 10H); ^13^C NMR (CDCl_3_, 100 MHz, 298 K) δ 171.2, 158.3, 153.2, 129.6, 129.3, 128.7, 114.3, 104.9, 104.3, 79.9, 74.0, 73.2, 70.2, 67.9, 62.7, 60.3, 57.9, 56.0, 32.6, 29.9, 29.6, 29.30, 29.27, 29.2, 28.4, 26.0, 25.9, 25.5, 25.4; HRMS–ESI–TOF (*m*/*z*): [M + Na]^+^ calcd for C_40_H_61_NO_8_Na^+^, 706.4289; found, 706.4295.

**Compound 4:** To the mixture of compound **3** (2.0 g, 2.92 mmol) and allylacetic acid (0.88 g, 8.77 mmol) in DCM (50 mL) was added EDCI (2.24 g, 11.68 mmol) and DMAP (0.36 g, 2.92 mmol) while cooling with an ice bath. After that the mixture was stirred overnight at room temperature and then washed the mixture with brine (3 × 50 mL). The organic layer was dried over anhydrous sodium sulfate. The combined organic layer was evaporated, and the residue was purified via column chromatography (SiO_2_, PE/EA = 15:1) to give compound **4** (1.97 g, 88%) as a yellow oil. ^1^H NMR (CDCl_3_, 400 MHz, 298 K) δ 7.21–7.03 (m, 2H), 6.84 (d, *J* = 8.4 Hz, 2H), 6.38 (d, *J* = 22.8 Hz, 2H), 5.87–5.76 (m, 1H), 5.09–4.95 (m, 2H), 4.29 (dd, *J* = 32.0 Hz, *J* = 16.4 Hz, 4H), 4.13 (d, *J* = 2.4 Hz, 2H), 4.08 (t, *J* = 6.8 Hz, 2H), 3.94 (t, *J* = 6.4 Hz, 4H), 3.78 (s, 6H), 3.50 (t, *J* = 6.8 Hz, 2H), 5.18–2.33 (m, 5H), 1.81–1.70 (m, 4H), 1.68–1.39 (m, 21H), 1.34–1.28 (m, 8H); ^13^C NMR (CDCl_3_, 100 MHz, 298 K) δ 173.1, 158.4, 155.9, 153.4, 136.7, 129.7, 129.3, 128.7, 115.4, 114.4, 104.9, 104.3, 80.0, 74.0, 73.1, 70.4, 70.2, 67.9, 64.4, 57.9, 56.0, 33.5, 29.9, 29.4, 29.33, 29.30, 29.2, 28.8, 28.6, 28.4; HRMS–ESI–TOF (*m*/z): [M + Na]^+^ calcd for C_45_H_67_NO_9_Na^+^, 788.4708; found, 788.4732.

**Compound 5:** TFA (1.9 mL, 25.72 mmol) was added to a solution of the product **4** (1.97 g, 25.72 mmol) in dichloromethane (50 mL) and the mixture was stirred for 10 h at room temperature. The organic solvent was evaporated under reduced pressure to get a yellow solid, which was dissolved in MeOH (50 mL) and 20 mL saturated aqueous solution of NH_4_PF_6_ was added. After stirring for 5 h at room temperature, the mixture was diluted with CH_2_Cl_2_ (100 mL), the organic layer was separated and evaporated under reduced pressure to get the crude product, which was purified by column chromatography (SiO_2_, CH_2_Cl_2_/MeOH = 50:1) to afford product **5** (1.67 g, 80%) as a yellow solid. ^1^H NMR (CDCl_3_, 400 MHz, 298 K) δ 7.26 (d, *J* = 8.8 Hz, 2H), 6.84 (d, *J* = 8.8 Hz, 2H), 6.57 (s, 2H), 5.90–5.74 (m, 1H), 5.13–4.89 (m, 2H), 4.13 (d, *J* = 2.4 Hz, 2H), 4.08 (t, *J* = 6.8 Hz, 2H), 3.97–3.88 (m, 4H), 3.84 (s, 6H), 3.74 (d, *J* = 6.0 Hz, 4H), 3.51 (t, *J* = 6.4 Hz, 2H), 2.48–2.32 (m, 5H), 1.81–1.70 (m, 4H), 1.69–1.55 (m, 4H), 1.53–1.38 (m, 6H), 1.36–1.28 (m, 10H); ^13^C NMR (CDCl_3_, 100 MHz, 298 K) δ 173.3, 160.3, 153.4, 136.8, 136.5, 131.4, 125.2, 121.4, 115.4, 115.0, 106.6, 79.9, 74.1, 73.1, 70.2, 68.0, 64.3, 57.9, 56.0, 51.6, 51.2, 33.4, 29.43, 29.38, 29.30, 29.28, 29.1, 28.8, 28.4, 26.0, 25.9, 25.5, 25.1; HRMS–ESI–TOF (*m*/*z*): [M − PF_6_^−^]^+^ calcd for C_40_H_60_NO_7_^+^, 666.4364; found, 666.4361.

**Compound R1:** A mixture of **5** (300 mg, 0.370 mmol) and crown ether **9** (414 mg, 0.739 mmol) was dissolved in dry CH_2_Cl_2_ (10 mL) and stirred for 0.5 h at room temperature. Then compound **8** (475 mg, 0.555 mmol) and [Cu(CH_3_CN)_4_]PF_6_ (138 mg, 0.370 mmol) were added to the solution. The mixture was stirred for two days under an argon atmosphere at room temperature. After removal of the solvent, the residue was purified via column chromatography (SiO_2_, CH_2_Cl_2_/MeOH = 80:1) to give compound **R1** (495 mg, 60%) as a yellow solid. ^1^H NMR (CDCl_3_, 400 MHz, 298 K) δ 7.61–7.42 (m, 3H), 7.33 (t *J* = 4.8 Hz, 1H), 7.16 (d *J* = 8.8 Hz, 2H), 7.06 (s, 2H), 6.91–6.83 (m, 3H), 6.82–6.72 (m, 4H), 6.63 (d *J* = 8.8 Hz, 2H), 6.58–6.45 (m, 3H), 5.87–5.71 (m, 2H), 5.09–4.91 (m, 6H), 4.68–4.53 (m, 4H), 4.51–4.40 (m, 2H), 4.31 (t, *J* = 7.2 Hz, 2H), 4.13–4.04 (m, 10H), 4.00–3.93 (m, 6H), 3.85–3.70 (m, 12H), 3.56 (s, 6H), 3.53–3.46 (m, 8H), 3.23 (dd, *J* = 12.8 Hz, *J* = 6.8 Hz, 2H), 2.46–2.32 (m, 8H), 2.07–1.99 (m, 2H), 1.92–1.83 (m, 2H), 1.78–1.58 (m, 12H), 1.51–1.36 (m, 14H), 1.31–1.22 (m, 62H), 0.86 (t, *J* = 6.4 Hz, 9H); ^13^C NMR (CDCl_3_, 100 MHz, 298 K) δ 173.1, 172.8, 169.2, 167.5, 159.8, 153.4, 153.0, 147.3, 147.1, 145.2, 141.0, 137.4, 136.6, 136.5, 130.6, 129.4, 128.3, 127.4, 122.8, 122.5, 121.8, 121.7, 115.5, 115.4, 114.4, 112.8, 112.33, 112.26, 105.7, 105.5, 73.4, 73.2, 70.8, 70.4, 70.1, 70.0, 69.1, 68.1, 68.0, 67.9, 65.8, 64.4, 64.1, 55.9, 52.3, 52.1, 50.0, 43.8, 39.1, 33.5, 33.4, 31.9, 30.3, 29.9, 29.7, 29.64, 29.62, 29.58, 29.53, 29.36, 29.32, 29.30, 29.27, 29.1, 28.9, 28.8, 28.7, 28.5, 26.03, 26.01, 25.9, 25.8, 25.7, 25.6, 25.4, 22.6, 14.1; HRMS–ESI–TOF (*m*/*z*): [M −PF_6_^−^]^+^ calcd for C_121_H_193_N_6_O_22_^+^, 2083.4196; found, 2083.3862.

## Supporting Information

File 1 ^1^H, ^13^C NMR spectra and HRESI mass spectra of compounds **3**, **4**, **5**, **8** and [2]rotaxane **R1**.
