# Supplementary material for: A switchable [2]rotaxane with two active alkenyl groups
Source: Beilstein J Org Chem. 2018 Aug 8;14:2074–81. doi: 10.3762/bjoc.14.181 (PMC6122368; doi:10.3762/bjoc.14.181)
Supplement: File 1 — 1H, 13C NMR spectra and HRESI mass spectra of compounds 3, 4, 5, 8 and [2]rotaxane R1. [file Beilstein_J_Org_Chem-14-2074-s001.pdf]

Supporting Information

for

A switchable [2]rotaxane with two active alkenyl groups

Xiu-Li Zheng, Rong-Rong Tao, Rui-Rui Gu, Wen-Zhi Wang, Da-Hui Qu*

Address: Key Laboratory for Advanced Materials and Institute of Fine Chemicals, School of Chemistry and Molecular Engineering, East China University of Science and Technology, 130 Meilong Road, Shanghai, 200237, China

Email: Da-Hui Qu* - dahui_qu@ecust.edu.cn

*Corresponding author

^{1}H, ^{13}C NMR spectra and HRESI mass spectra of compounds 3, 4, 5, 8 and [2]rotaxane R1

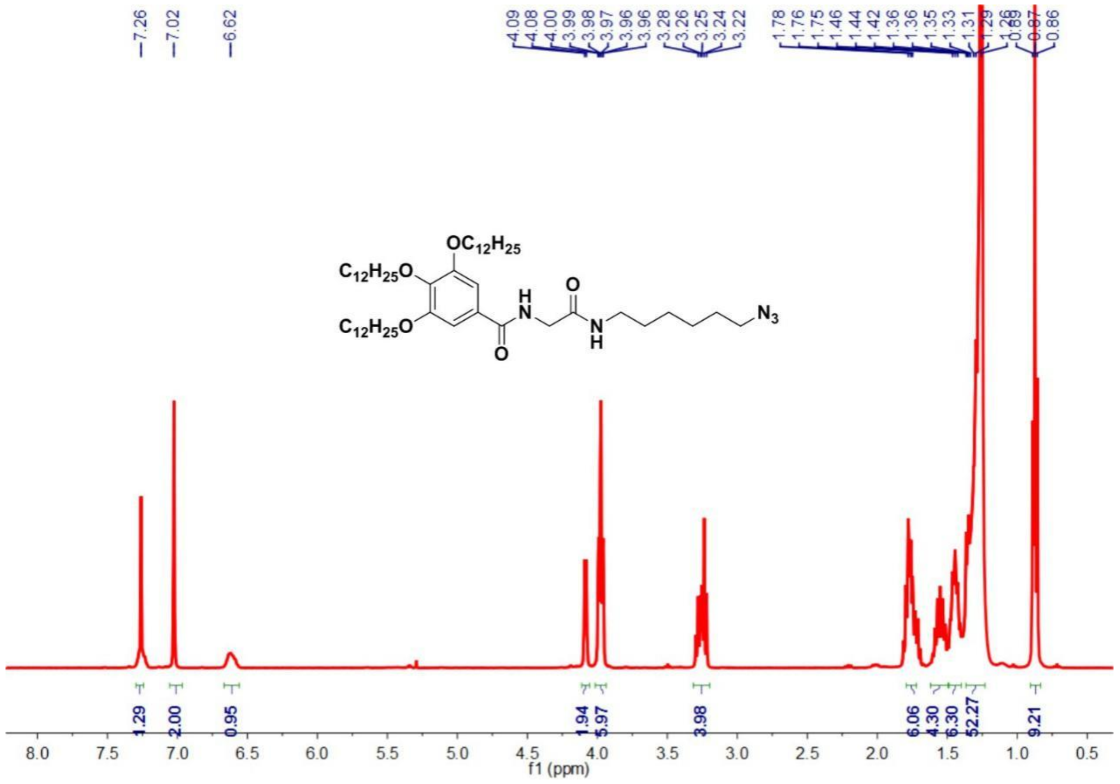

Figure S1: ^{1}H NMR spectrum of compound **8** (400 MHz, $CDCl_3$, 298 K).

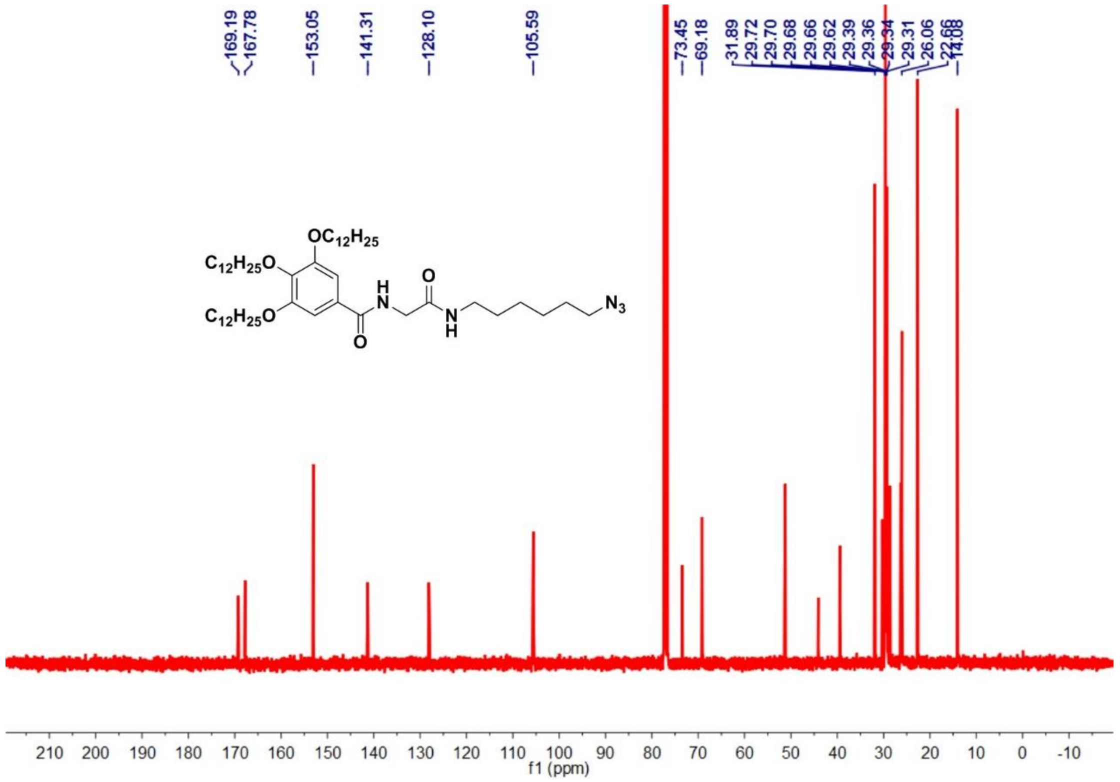

Figure S2: ^{13}C NMR spectrum of compound **8** (100 MHz, $CDCl_3$, 298 K).

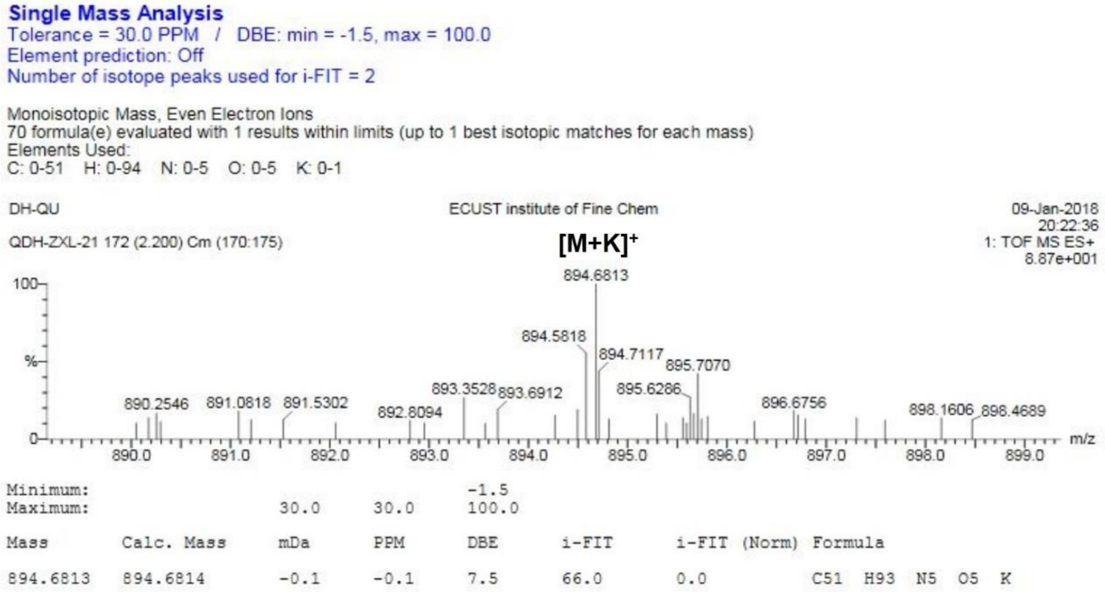

Figure S3: HRESI mass spectrum of compound **8**.

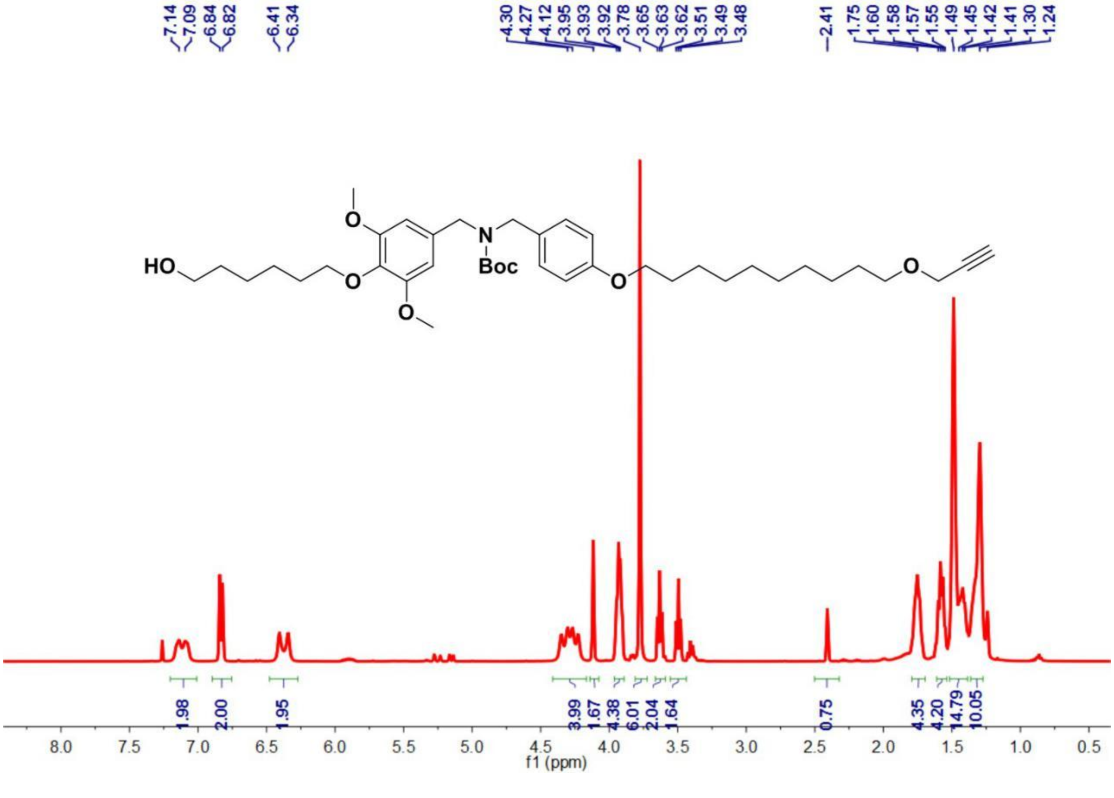

Figure S4: ^{1}H NMR spectrum of compound **3** (400 MHz, $CDCl_3$, 298 K).

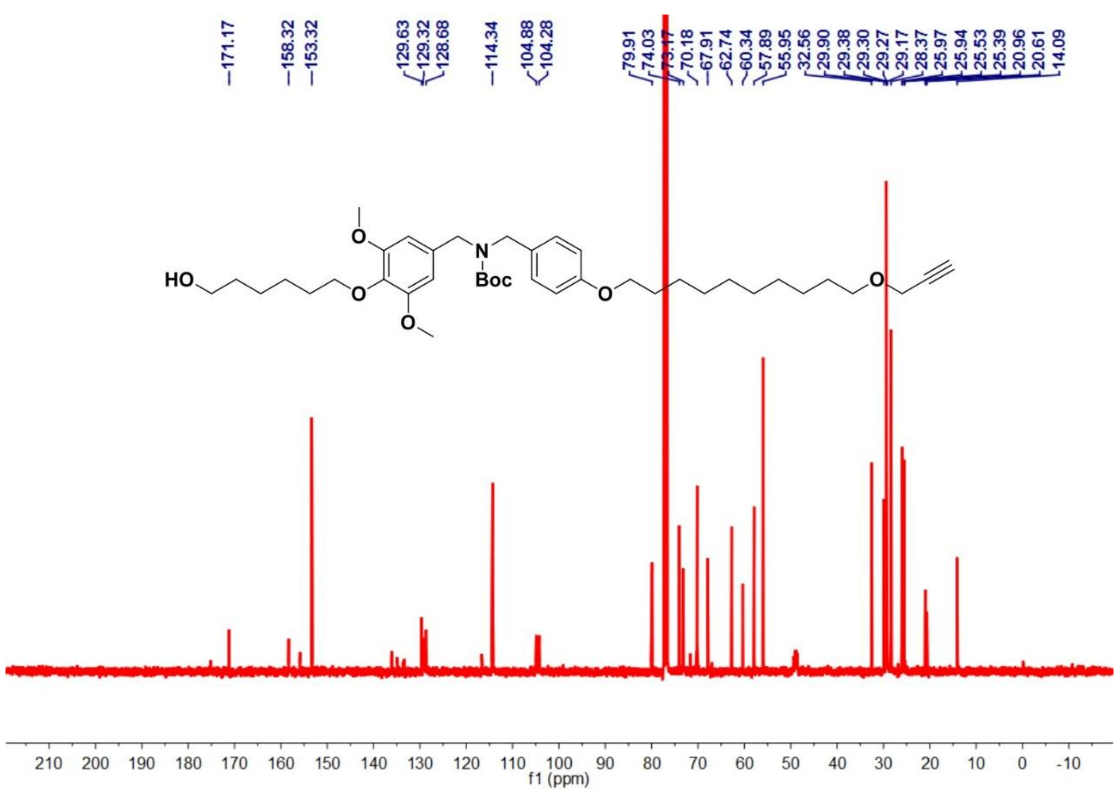

Figure S5: ^{13}C NMR spectrum of compound **3** (100 MHz, $CDCl_3$, 298 K).

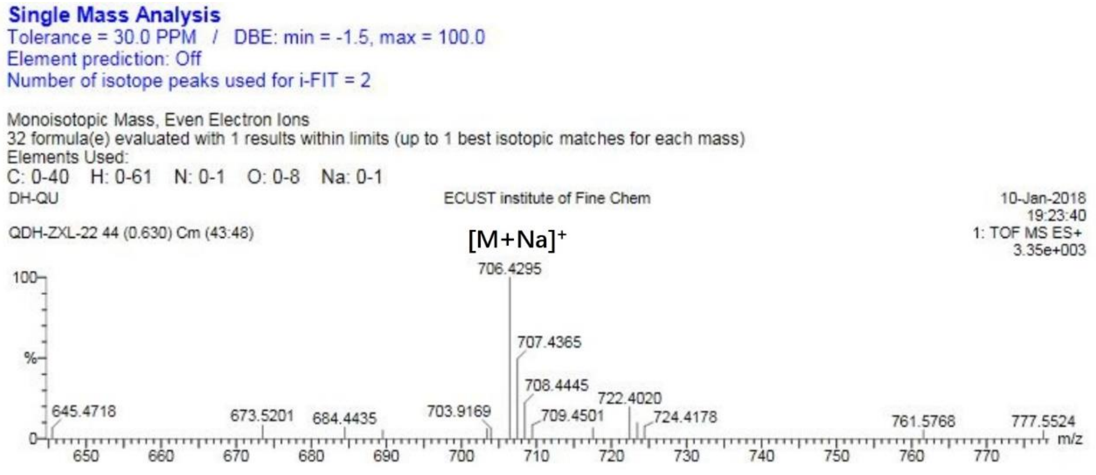

Minimum:				-1.5			
Maximum:		30.0	30.0	100.0			
Mass	Calc. Mass	mDa	PPM	DBE	i-FIT	i-FIT (Norm)	Formula
706.4295	706.4295	0.0	0.0	10.5	11.4	0.0	C40 H61 N O8 Na

Figure S6: HRESI mass spectrum of compound **3**.

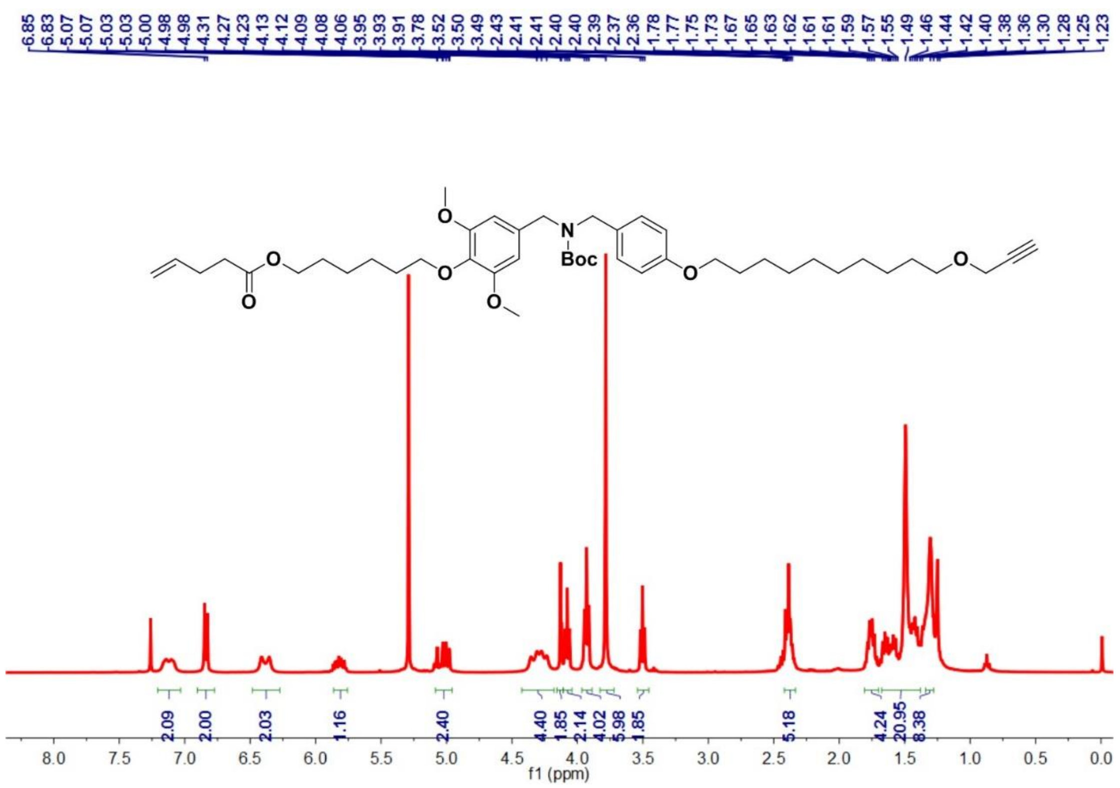

Figure S7: ^{1}H NMR spectrum of compound **4** (400 MHz, $CDCl_3$, 298 K).

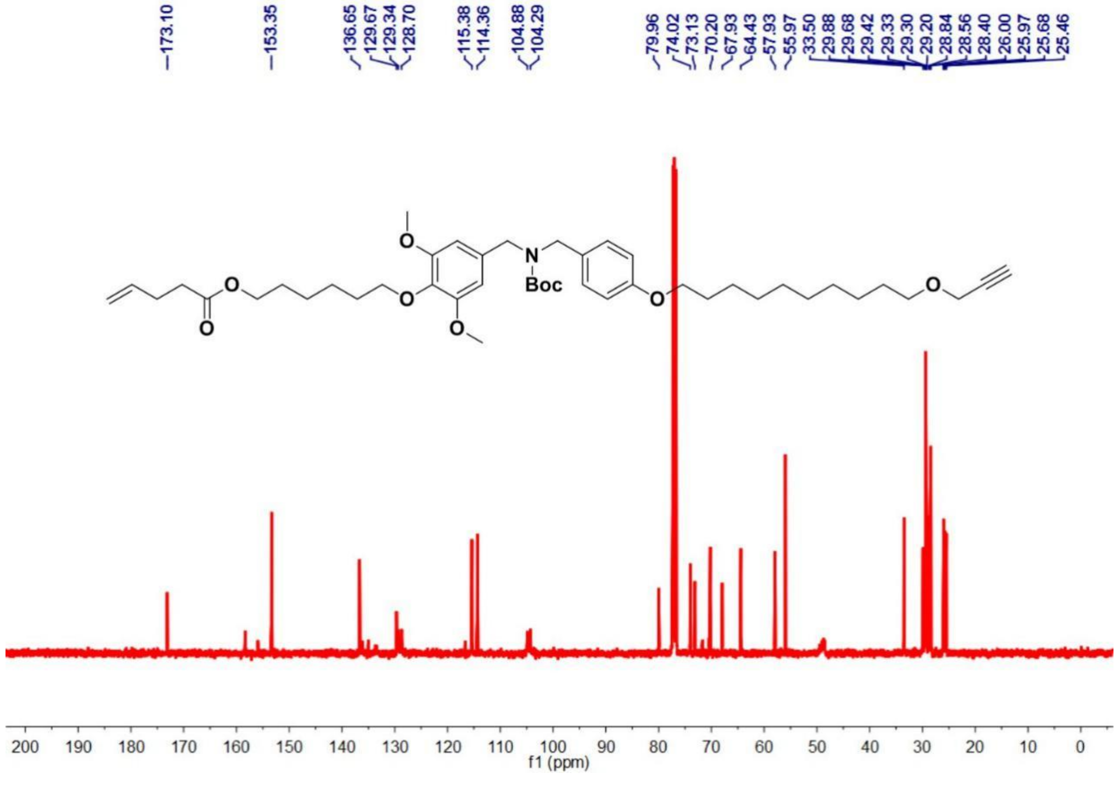

Figure S8: ^{13}C NMR spectrum of compound **4** (100 MHz, $CDCl_3$, 298 K).

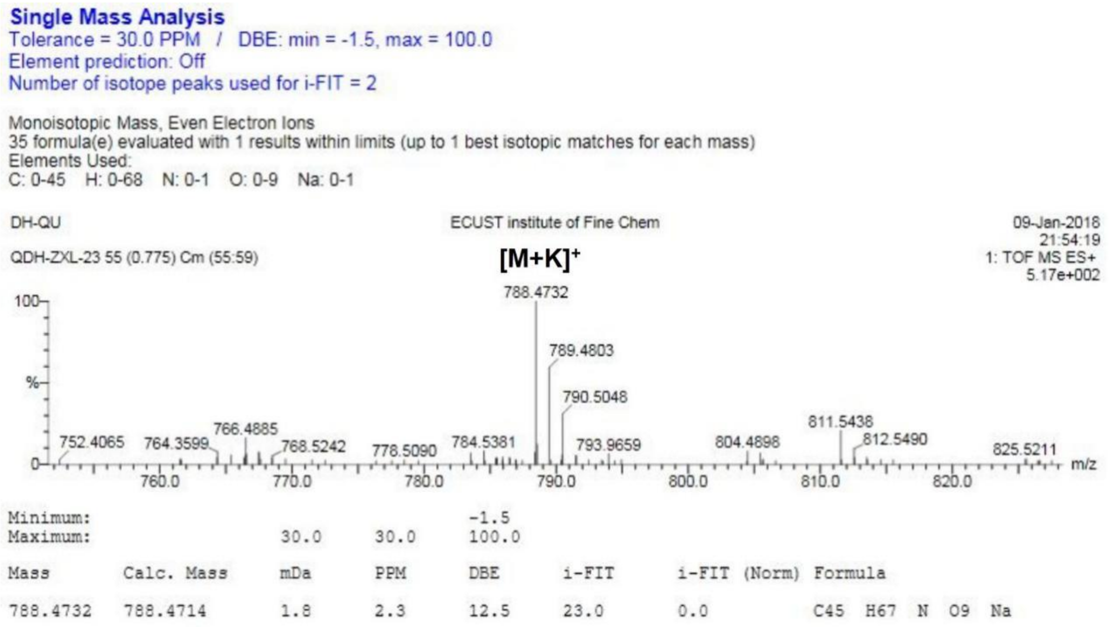

Figure S9: HRESI mass spectrum of compound **4**.

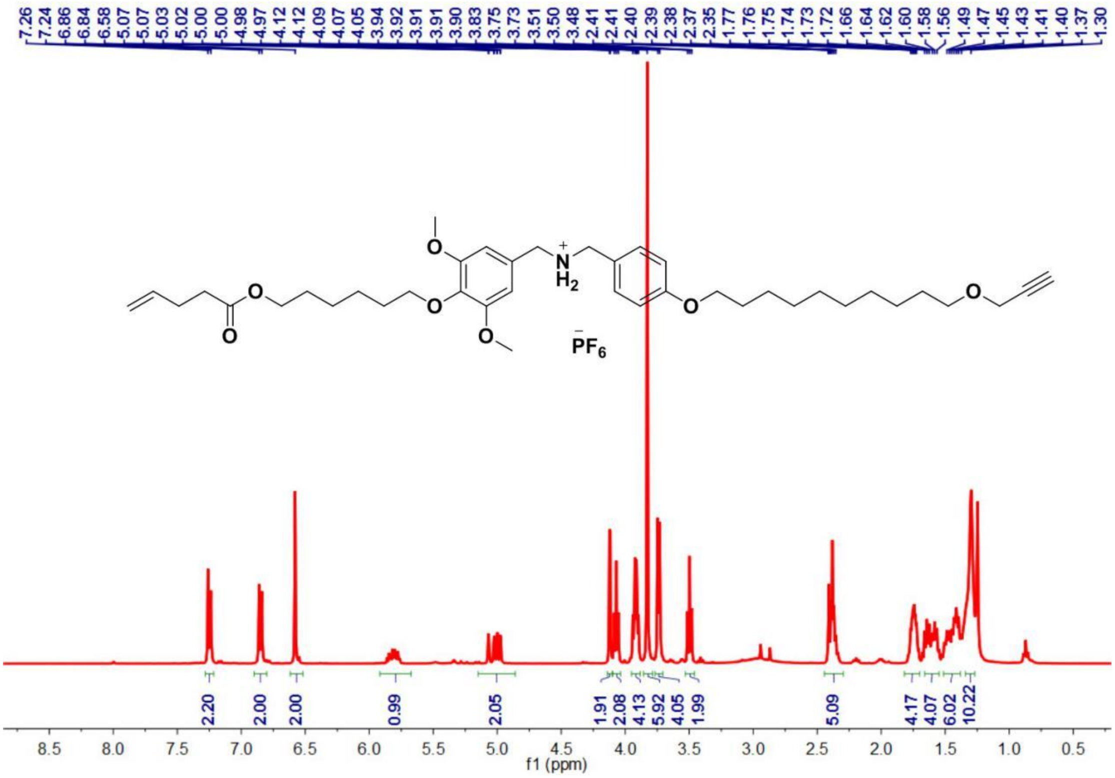

Figure S10: ^{1}H NMR spectrum of compound **5** (400 MHz, $CDCl_3$, 298 K).

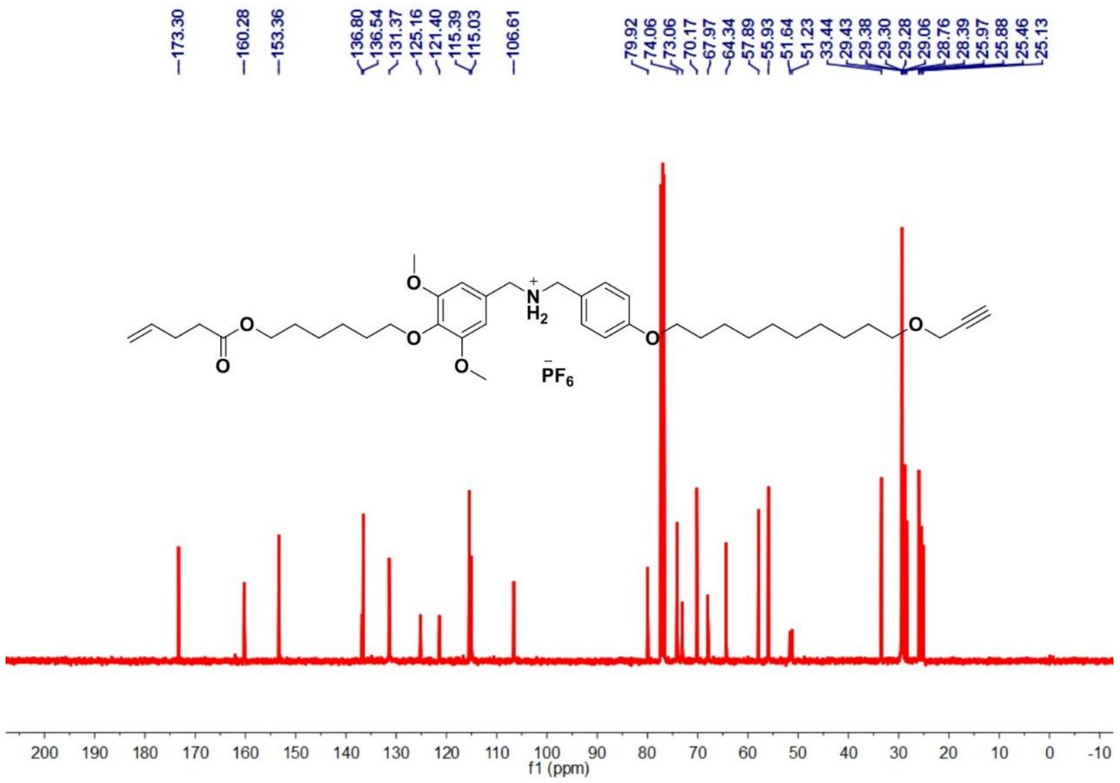

Figure S11: ^{13}C NMR spectrum of compound **5** (100 MHz, $CDCl_3$, 298 K).

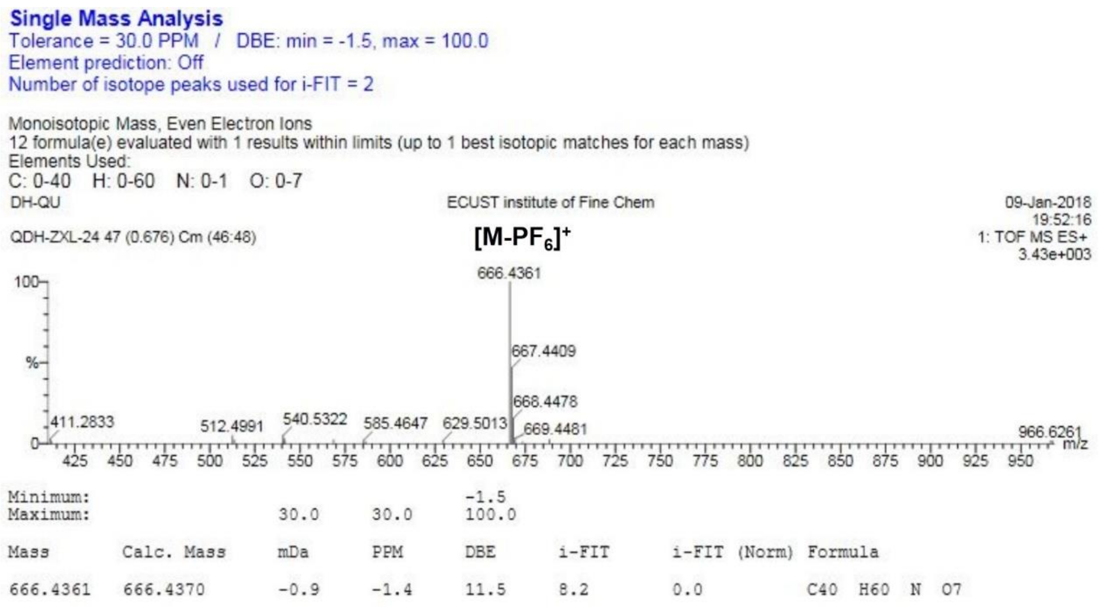

Figure S12: HRESI mass spectrum of compound **5**.

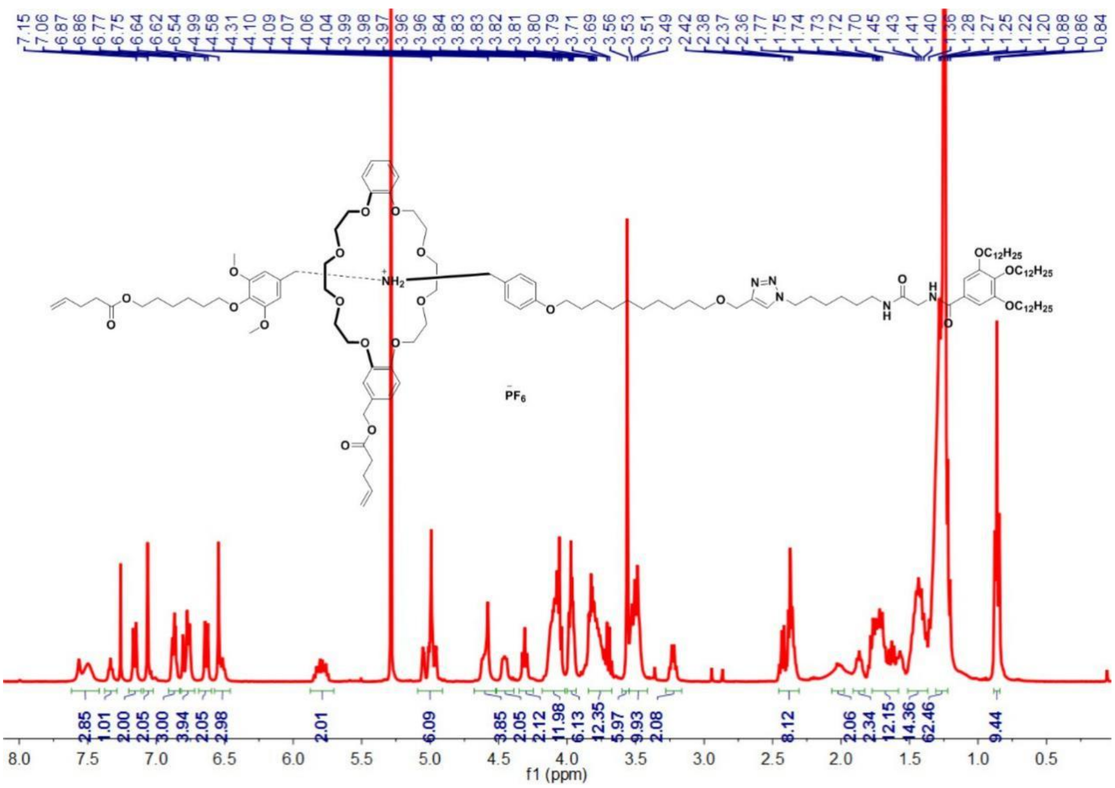

Figure S13: ^{1}H NMR spectrum of [2]rotaxane **R1** (400 MHz, $CDCl_3$, 298 K).

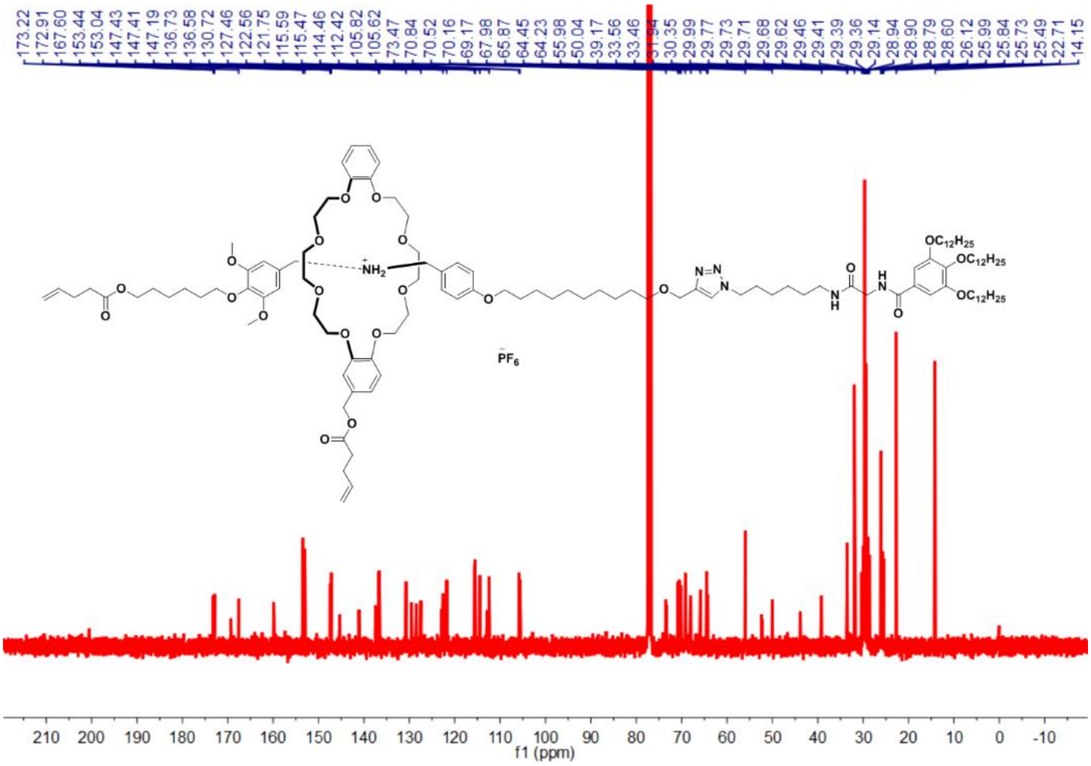

Figure S14: ^{13}C NMR spectrum of [2]rotaxane **R1** (100 MHz, $CDCl_3$, 298 K).

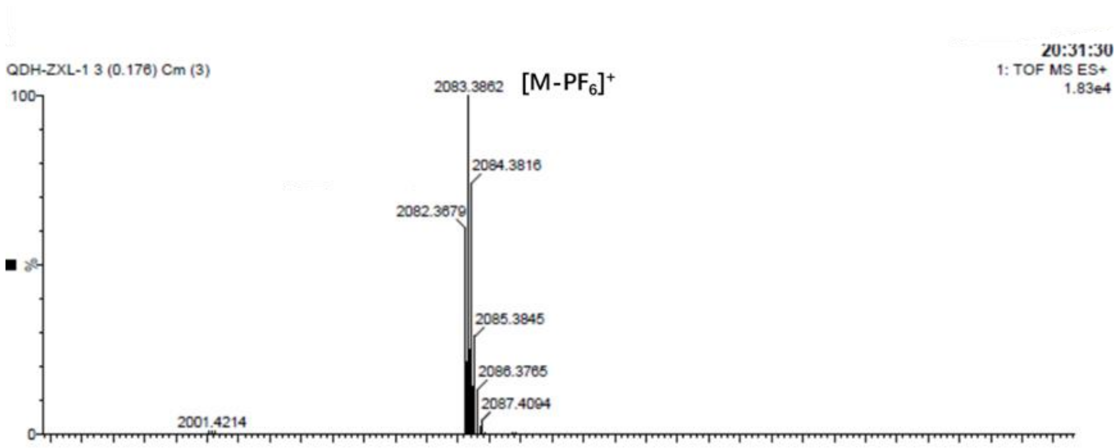

Figure S15: HRESI mass spectrum of [2]rotaxane **R1**.
